# Resolving homology in the face of shifting germ layer origins: Lessons from a major skull vault boundary

**DOI:** 10.7554/eLife.52814

**Published:** 2019-12-23

**Authors:** Camilla S Teng, Lionel Cavin, Robert E Maxson, Marcelo R Sánchez-Villagra, J Gage Crump

**Affiliations:** 1 Department of Stem Cell Biology and Regenerative MedicineUniversity of Southern CaliforniaLos AngelesUnited States; 2 Department of Biochemistry, Keck School of MedicineUniversity of Southern CaliforniaLos AngelesUnited States; 3 Department of Earth SciencesNatural History Museum of GenevaGenevaSwitzerland; 4 Paleontological Institute and MuseumUniversity of ZurichZurichSwitzerland; Max-Planck Institute for Evolutionary BiologyGermany; Max-Planck Institute for Evolutionary BiologyGermany

**Keywords:** skull, sutures, homology

## Abstract

The vertebrate skull varies widely in shape, accommodating diverse strategies of feeding and predation. The braincase is composed of several flat bones that meet at flexible joints called sutures. Nearly all vertebrates have a prominent ‘coronal’ suture that separates the front and back of the skull. This suture can develop entirely within mesoderm-derived tissue, neural crest-derived tissue, or at the boundary of the two. Recent paleontological findings and genetic insights in non-mammalian model organisms serve to revise fundamental knowledge on the development and evolution of this suture. Growing evidence supports a decoupling of the germ layer origins of the mesenchyme that forms the calvarial bones from inductive signaling that establishes discrete bone centers. Changes in these relationships facilitate skull evolution and may create susceptibility to disease. These concepts provide a general framework for approaching issues of homology in cases where germ layer origins have shifted during evolution.

## Introduction

**Figure 1.**
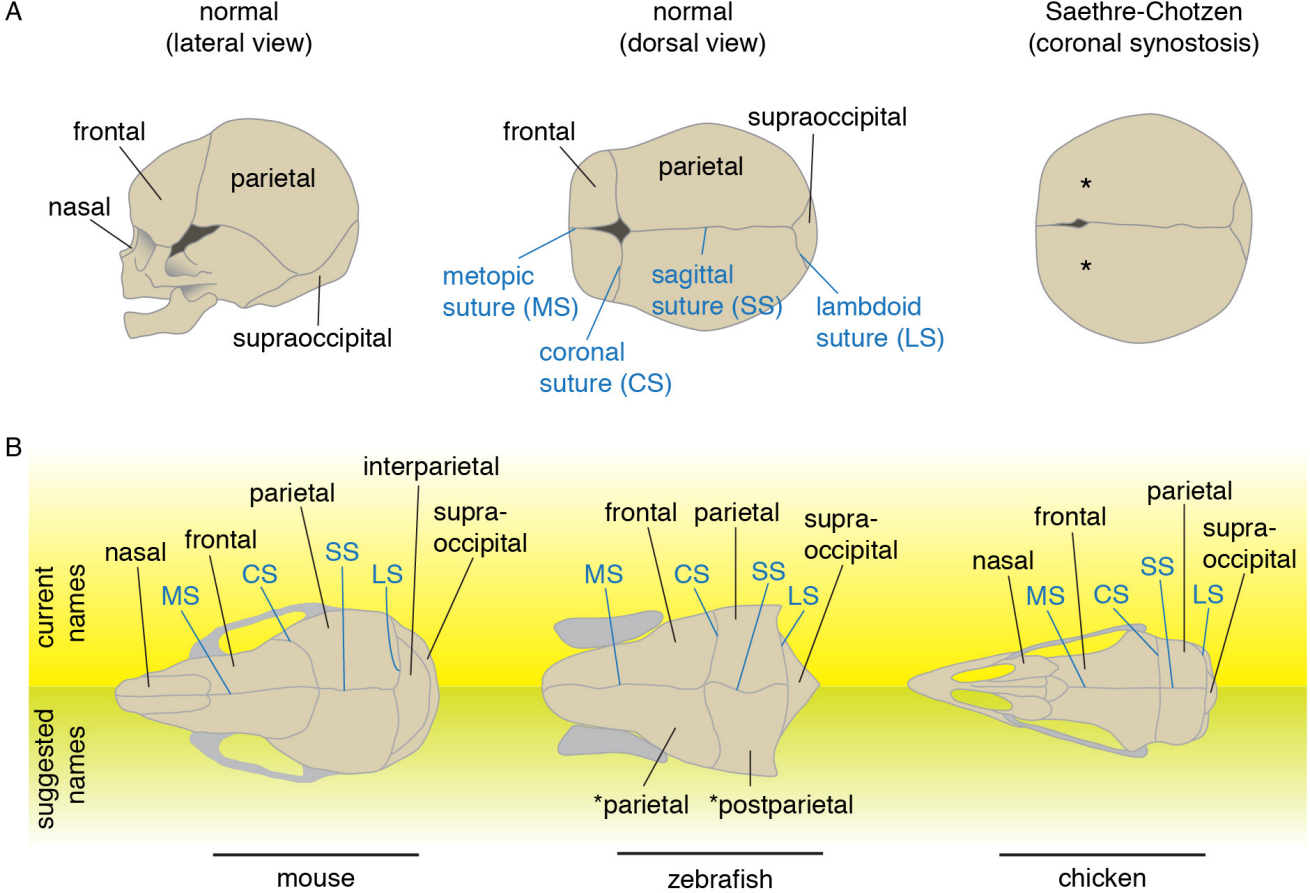
Schematics and nomenclature of major skull vault bones and sutures. (**A**) Diagrams of normal and synostotic human skulls. Bones are labeled in black and sutures in blue. Asterisks denote the missing coronal suture in Saethre-Chotzen Syndrome skulls. (**B**) Major skull vault bones and sutures in dorsal views (anterior to the left). Current names are listed on top, and suggested names for zebrafish are listed at bottom. Note that in the revised naming of zebrafish bones, the zebrafish coronal suture is analogous to the lambdoid suture of mouse as the murine interparietal is a fusion of postparietals and tabulars.

At the beginning of skull vault development, mesenchymal cells of either neural crest or mesoderm origin condense into nascent bone fields. Embryonic calvarial bones then grow through addition of new osteoblasts at their leading edges, until bone covers the majority of the roof of the head. Where bones meet, fibrous sutures form. These sutures serve as flexible attachment sites between bones, and house progenitor cells that allow further growth of the bones and hence growth of the skull and brain (Doro et al., 2017). Sutures also provide a source of osteoblasts for bone repair (Zhao et al., 2015). As defined for humans, major sutures include the coronal (interface of frontal and parietal bones), sagittal (parietal-parietal), metopic (frontal-frontal), and lambdoid (parietal-supraoccipital) (Figure 1A). In some continuously growing vertebrates (e.g. zebrafish), most if not all sutures remain patent throughout life (Cubbage and Mabee, 1996). In other tetrapods, including groups of archosaurs (Bailleul et al., 2016) and monotreme, marsupial, and placental mammals (Rager et al., 2014), sutures can be transient structures. For example, most sutures begin to close shortly after puberty in humans. Nonetheless, the general pattern of the major sutures is largely conserved across vertebrates, although gain or loss of specific sutures has been linked with evolutionary changes in skull morphology.

The premature loss of sutures during human fetal life is seen in craniosynostosis, a birth defect that occurs in approximately one in 2500 live births (Twigg and Wilkie, 2015). Without surgical intervention, altered skull growth may have an adverse effect on brain development. The most widely used treatment consists of surgically separating the fused bones to provide space for the growing brain. Unfortunately, re-fusion of the separated bones often necessitates additional surgeries. Given the high morbidity associated with such invasive surgeries, a better understanding of the developmental basis of pathological suture loss may bring better treatments to this condition. A number of genetic mutations have been found to cause craniosynostosis, largely in the context of a broader syndrome (Twigg and Wilkie, 2015). Many of these syndromic synostoses are highly specific to particular sutures, suggesting unique developmental sensitivities of each type of suture. Whereas the sagittal is most often affected in non-syndromic craniosynostosis, the coronal is most frequently perturbed in syndromic cases. The most common form of syndromic coronal synostosis is Saethre-Chotzen syndrome (Figure 1A), which is caused by heterozygous loss-of-function mutations in *TWIST1* or *TCF12* (el Ghouzzi et al., 1997; Howard et al., 1997; Sharma et al., 2013). Research resulting from the recent development of both mouse and zebrafish models for Saethre-Chotzen syndrome reveals deep conservation of the genetic programs underlying coronal suture development, despite dramatic shifts in the embryonic origins of the bones that abut this suture (Sharma et al., 2013; Teng et al., 2018).

We will discuss (i) paleontological and embryological evidence pertaining to the question of whether the coronal suture is homologous across vertebrate taxa, (ii) the mechanisms by which the coronal suture may be positioned in diverse taxa, and (iii) how new insights into the evolutionary and developmental history of the coronal suture may illuminate why this suture is particularly affected in syndromic synostoses. More broadly, while this review focuses on the skull, our synthesis addresses issues of how to conceptualize deep homology of vertebrate structures, i.e. how structures deriving from seemingly different embryonic tissues can share common and ancient sets of genetic programs.

## Diversity of skull bones and their sutures across extinct and living vertebrates

The mouse and zebrafish models belong to lineages that split from the human lineage circa 62 and 420 million years ago, respectively (Benton et al., 2015). During these long time-intervals, the pattern of the skull vault has diverged substantially from the common ancestors of rodents and primates on one side, and from the two major lineages of bony fishes, the sarcopterygians (lobe-finned fish) and the actinopterygians (ray-finned fish). Taxonomic identification and phylogenetic reconstructions based on the vertebrate fossil record rest in large part on deciphering the transformation of the skull roof pattern, which is often well preserved in fossils. Consequently, there is a great deal of morphological information about the bones of the skull roof in extant and fossil vertebrates (see Table 1 for major clades discussed). Recognition of homologies is, however, based solely on the topological arrangement of the bones; developmental and genetic data can rarely be used as evidence (Wagner, 2007).

**Table 1.** Systematic classification of the taxa quoted in the text. Dialipina* is also resolved as a stem osteichthyan in recent phylogenetic studies.

Systematics			
Osteichthyes (*bony fish and four-limbed animals*)			
Stem Osteichthyes			*Guiyu*
Actinopterygii (*ray-finned fish*)	Stem		Dialipina*
			*Meemannia*
			*Raynerius*
			*Lingulalepis*
			*Cheirolepis*
	Holostei (*gars and bowfin*)		*Amia* (bowfin)
	Teleostei (*teleosts*)		*Danio* (zebrafish)
Sarcopterygii (*lobe-finned fish and* *four-limbed animals*)	Actinistia (*coelacanth*)		*Latimeria* (coelacanth)
	Dipnoi (*lungfish*)		*Powichthys*
	Tetrapodomorpha (*four-limbed animals*)	Stem	*Eusthenopteron*
			*Panderichthys*
			*Tiktaalik*
			*Ichthyostega*
	Amphibia (*amphibians*)	Urodela (*salamanders, newts*)	*Ambystoma* (mole salamander)
		Anura (*frogs*)	*Xenopus* (clawed frog)
	Synapsida	Stem	*Dimetrodon*
		Mammalia (*mammals*)	*Mus* (mouse)
	Diapsida	Stem	*Orovenator*
		Aves (*birds*)	*Gallus* (chicken)

Names of the ‘fish’ bones were originally based on mammalian terminology, itself derived from human terminology (Hanken and Hall, 1993). The orbit (eye socket) was regarded as a landmark, and the paired bones between the orbits named ‘frontal bones.’ It is widely accepted that in order to reflect historical identity, the recognition of homologous bones should be based on evolutionary continuity, the principle that features of an organism exist in continuity with those of related organisms (Wagner, 2007; Borgen, 1983). Based on this criterion, Schultze (2008a), summing up almost a century of discussion, argued that frontal bones are a newly appearing feature of tetrapodomorphs (Schultze, 2008a), a lineage of sarcopterygians including modern tetrapods and their ancestral relatives but distinct from the lungfishes. What was originally called the ‘frontal’ in actinopterygians, based on the location of that bone between the orbits, is actually the parietal by evolutionary continuity with sarcopterygians (Figure 1B.

**Figure 2.**
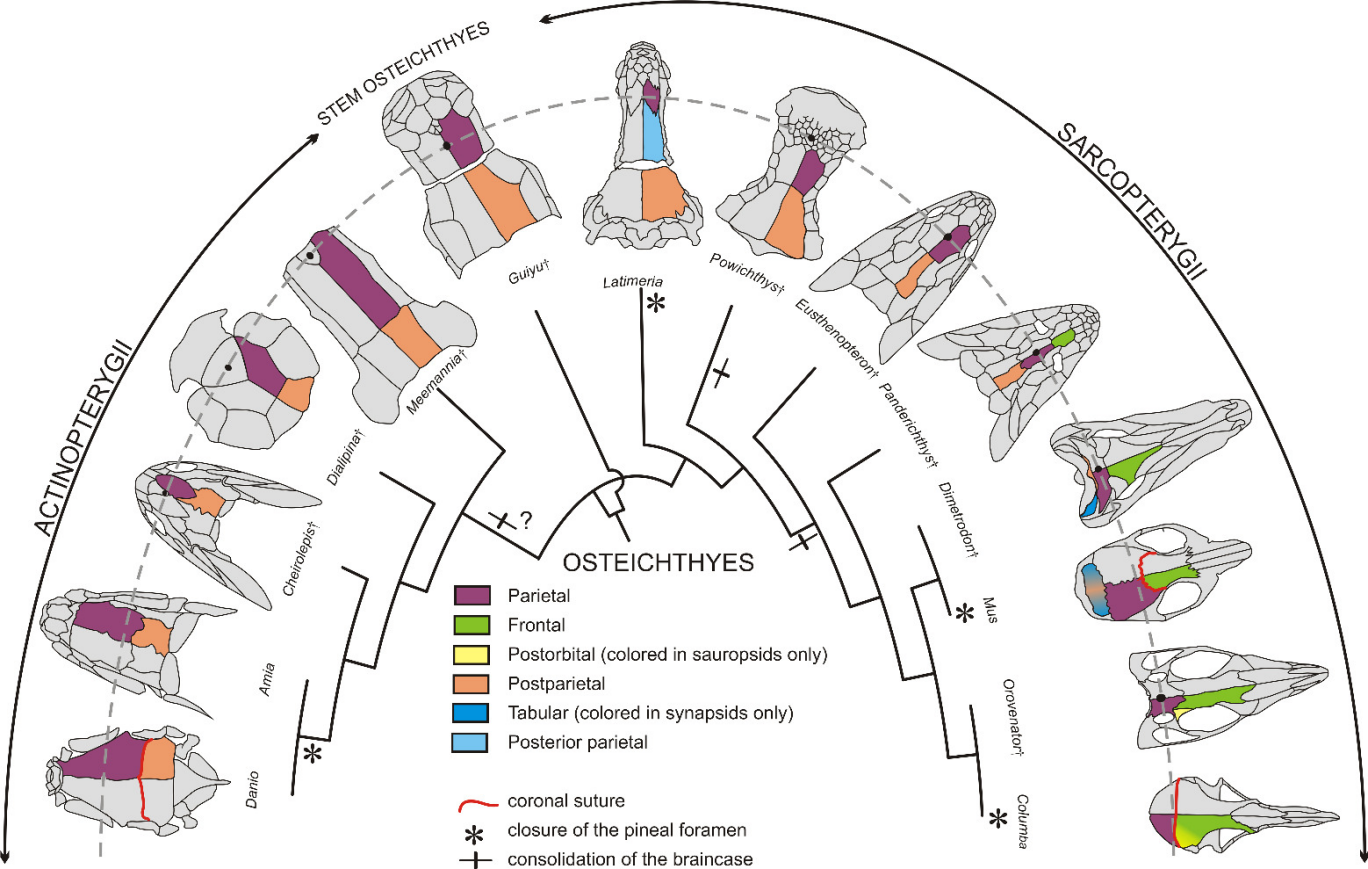
Evolution of the coronal suture and associated skull bones. Skull bones associated to the ‘coronal suture’ are mapped on a phylogeny of selected extant and extinct osteichthyans. On one side of each skull, bones are color-coded based on deductions from evolutionary continuity – for example the actinopterygian ‘parietal’ is what was formerly referred to as the ‘frontal’ in these species. The pineal foramen is shown by a black dot. The currently accepted coronal suture is shown for *Danio* (zebrafish), *Mus* (mouse), and *Columba* (pigeon). Daggers denote extinct species represented only by fossils.

What does the fossil record teach us about homology of the coronal suture and its associated calvarial bones across vertebrates? The coronal suture is defined in mammals as occurring between the parietal and frontal bones. Proper analysis therefore requires examination of the arrangement of the parietal with its adjacent anterior bones, and conversely the arrangement of the frontal with its adjacent posterior bones in the two groups of jawed vertebrates (sarcopterygians and actinopterygians). Here the search for independent topographical landmarks is paramount. The pineal foramen is one such landmark based on its position in the roof of the diencephalon, thus allowing placement of particular bones relative to the brain. It has been used to establish bone homologies between piscine sarcopterygians and basal tetrapods (for instance Borgen, 1983; Jarvick, 1966; Westoll, 1938). The pineal foramen was ancestrally present in osteichthyans, but was lost independently along the actinopterygian lineage, and several times within the sarcopterygian clade (e.g. along coelacanth, lungfish, bird, and mammalian lineages; see Figure 2). Developmentally, the pineal organ arises from an evagination of the third ventricle of the diencephalon, just posterior to the telencephalon. As the telencephalon-diencephalon boundary appears to coincide with the boundary of neural crest and mesoderm germ layer contribution to the skull bones in rodents (Papp et al., 2015) and zebrafish (Blümel et al., 2019), the pineal foramen, being posterior to this boundary, would be a landmark for the mesoderm-derived bone (i.e. the parietal in mammals). The location of the pineal foramen could thus serve to potentially identify the parietal homolog in fossil and extant osteichthyans, although more experimental data in extant animals are required to further substantiate this anatomical correlation.

Stem osteichthyans from the Silurian and Early Devonian, in other words the precursors to both actinopterygians and sarcopterygians, include the ‘psarolepids’ clade and *Lingulalepis* (Clement et al., 2018; Lu et al., 2017). In the ‘psarolepid’ *Guiyu* from the Silurian of China, for instance, what we now consider as parietals (previously called frontals) are sutured anteriorly to the nasal bone, with several postrostrals intercalating at the juncture of the paired parietals and nasals (Figure 2). The pineal foramen opens on the sagittal suture between the parietals and has no contact with the postrostral bones; this position of the pineal foramen is a major reason we consider these bones to be parietals and not frontals (Qiao and Zhu, 2010). In the basal actinopterygian *Raynerius*, the pineal foramen also opens between a pair of bones that we would hence term parietals, despite these being previously referred to as ‘frontals’ based on conventional actinopterygian terminology (Giles et al., 2015). In basal Devonian actinopterygians, such as *Meemannia*, *Dialipina* (also regarded as a stem osteichthyan in some recent phylogenies Clement et al., 2018), and *Cheirolepis*, the parietals suture medially to a postrostral bone at their anterior ends, with this postrostral containing the pineal foramen (called the pineal plate) (Figure 2) (Arratia and Cloutier, 1996; Mickle, 2015). Again, the location of the pineal foramen would identify the articulating bones as parietals and not frontals as previously thought. In teleosteans (the largest class of actinopterygians), the rostral bones are fused to the ethmoid endoskeleton, and the dermal component is no longer distinguishable in the more derived teleosts (Patterson, 1975). In these fishes, such as the zebrafish, the bones of the skull roof located anterior to the parietals (previously referred to as frontals) are the nasals (see Figures 1 and 2 for revised terminology). The nasals may touch the parietals or rest on them, but they do not attach to them through a fibrous suture. It should be noted that actinopterygians form about half of the vertebrate taxic diversity and display a very high level of morphological disparity, thus making it challenging to bring support for the homology of the actinopterygian ‘frontal’ with the sarcopterygian ‘parietal’ for every major clade. For instance, in gars the skull pattern is highly derived. In these fishes, the snout is elongated and the parietal bone has shifted anteriorly and does not cover the anterior part of the brain (Grande, 2010). The bone has no relationship with the telencephalon-diencephalon boundary, at least in the adult (deep ontogenetic modifications may affect the organization of this part of the skull, as in *Latimeria* discussed below). In extinct holosteans, however, such as in *Macrosemimimus toombsi*, the arrangement of the skull roofing bone with the underlying braincase is comparable to the situation in other actinopterygians (Patterson, 1975). It is thus reasonable to assume that the ‘frontal’ of actinoptergyians may be generally homologous to the ‘parietal’ of sarcopterygians, though this remains open to debate.

In the sarcopterygian lineage, containing the mouse and the human, basal forms of Dipnomorpha (lungfishes and their relatives such as *Powichthys* from the Devonian of Canada and Spitsbergen) have paired parietals connected anteriorly to a mosaic of postrostrals. In these fishes, the pineal foramen is located in the middle of the postrostrals, thus placing the parietals even further posterior than in the stem osteichthyan *Guiyu* (Clement and Janvier, 2004) (Figure 2). In the basal tetrapodomorph *Eusthenopteron,* from the Devonian of Canada, bones anterior to the parietals are a medial postrostral and two lateral series of nasals. The pineal foramen opens between the parietals, similar to the stem osteichthyan condition (Westoll, 1937). A trend in basal tetrapods is the elongation of the snout, which is associated with the emergence of a pair of bones corresponding to what have traditionally been called ‘frontals’ in the mammalian literature (Schultze, 2008a). The frontal appears in the Devonian at some stage along the stem-tetrapod lineage, between the *Eusthenopteron* and the *Panderichthys* clades (Clack, 2012) (Figure 2). Bones anterior to the newly formed frontals are postrostral bone(s) in the earliest tetrapodomorphs (*Tiktaalik*, *Panderichthys*), with the paired nasals contacting each other in the midline in *Ichthyostega* and more crownward taxa, including the amniotes (Ahlberg et al., 2008). In basal tetrapodomorphs possessing a frontal (e.g. *Panderichthys*), the suture between this bone and the parietal, which corresponds to the coronal suture, is situated at the level of the orbit. The coronal suture then shifted posteriorly relative to the orbits in later tetrapods (Schultze and Arsenault, 1985). Nonetheless, in tetrapodomorphs in which the pineal foramen is still visible, it is commonly located between what are called the parietal bones, despite shifts in their relation to the orbits along the tetrapod lineage. In summary, at least in reference to the pineal foramen, the parietal of sarcopterygians would be homologous to the ‘frontal’ of stem osteichthyans and actinopterygians, thus justifying renaming it a ‘parietal’ in the latter based on evolutionary continuity (Figure 2). In tetrapodomorphs, a new bone, the true frontal, intercalates between the parietal and the postrostral(s). The new frontal bone corresponds either to a neoformation or to the division of the parietal bone of basal osteichthyans into parietal and frontal bones. In the latter case, we hypothesize that the tetrapod frontal corresponds to the part of the ancestral parietal with a neural crest origin, with the parietal having exclusively a mesodermal origin (with exceptions, such as the African clawed frog, see below).

In light of these evolutionary continuity-driven analyses regarding the homology of calvarial bones, we can update the current interpretation on the ancestral relationships of cranial sutures from different clades. In the mouse, the posterior end of the parietal sutures with the interparietal bone (itself a fusion of medial postparietal and lateral tabular bones Koyabu et al., 2012). If we interpret the actinopterygian ‘frontal’ as the equivalent of the mammalian ‘parietal’, then evolutionary continuity would dictate that what is called the ‘parietal’ in actinopterygians is homologous to the mammalian ‘postparietal’ (incorporation of the tabulars into the interparietal occurred later along the amniote lineage). As the parietal-interparietal joint of mouse is the lambdoid suture, the actinopterygian coronal suture being at a parietal-postparietal interface would therefore be homologous to the mammalian lambdoid suture. However, fossils show variation of how the parietal sutures with posterior bones in the evolutionary history of osteichthyans. Basal sarcopterygians, and possibly stem osteichthyans (Lu et al., 2017), had a braincase composed of two bones articulated through an intracranial joint – a hinge with considerable flexibility compared to standard sutures. The bone anterior to the intracranial joint is the parietal, as defined by the pineal foramen landmark, and the bone posterior to the intracranial joint is the postparietal. The only living vertebrate with an intracranial joint is the coelacanth *Latimeria*. In *Latimeria*, as in most extinct coelacanths, there are two pairs of parietals roofing the anterior part of the braincase (Forey, 1998). Recent analysis of coelacanth fetuses (Dutel et al., 2019) has revealed that the pineal complex, not visible on the surface of the brain (Hafeez and Merhige, 1977), and the telencephalon-diencephalon boundary are located at the level of the anterior parietal, according to Forey (1998), suggesting homology with the parietal of other osteichthyans. We therefore suggest that intercalation of a new bone (the posterior parietal) along the coelacanth lineage shifted the intracranial joint from a ‘parietal’-‘postparietal’ interface (as with the zebrafish ‘coronal’ and mouse ‘lambdoid’ suture) to a ‘posterior parietal’-‘postparietal’ interface. This acquisition of a new ‘posterior parietal’ bone in the coelacanth lineage is analogous to the gain of a new ‘frontal’ bone in tetrapodomorphs. Closure of the intracranial joint and suturing of the parietal with the postparietal occurred several times in the evolutionary history of osteichthyans, possibly at the base of the actinopterygian lineage, and certainly along the lungfish and tetrapodomorph lineages (Lu et al., 2017) (Figure 2). As a consequence, acquisition of the mammalian lambdoid and zebrafish coronal sutures would reflect convergent evolution, although their positioning at a common parietal-postparietal interface could still reflect the common developmental processes discussed below.

It is thus clear that the topology of calvarial bones and their suture boundaries have undergone substantial changes even in closely related groups of fishes. We can infer from fossil data that the parietal is a conserved structure across taxa, while the true frontal is a novel bone evolved in the tetrapodomorph lineage. Thus, the tetrapod coronal suture, as defined by a frontal-parietal interface, would be non-existent in actinopterygians including the zebrafish. Instead, the ‘coronal suture’ of these species would be analogous to the lambdoid suture of mouse. As homology is typically defined through common ancestry and embryonic tissue origin, examination of the latter criterion may be revealing. In the following sections, we discuss what developmental mechanisms inform us about the homology between the mouse and zebrafish coronal sutures.

## Embryological analysis of germ layer contributions to skull bone boundaries

**Figure 3.**
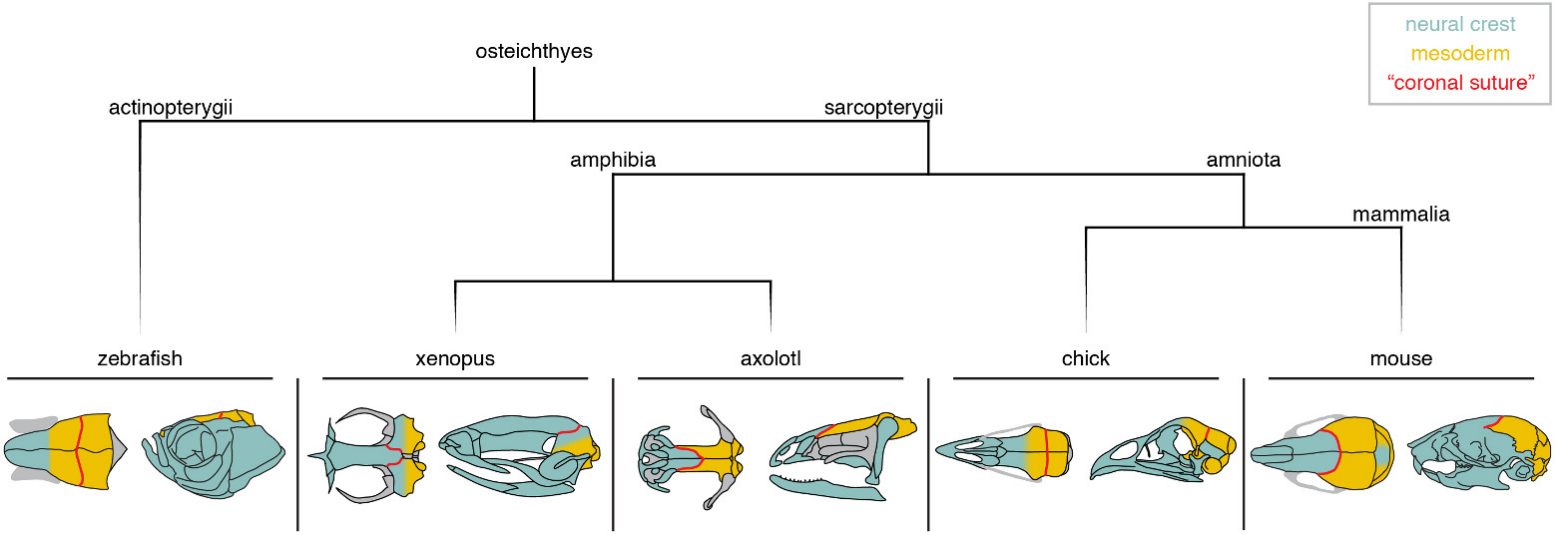
Neural crest-mesoderm boundaries relative to the coronal suture. Schematics depict neural crest (teal) and mesoderm (yellow) contributions to skull bones of lineage-traced model organisms. Red line indicates the position of the suture commonly referred to as the ‘coronal suture’ in each respective species, though this may not be reflective of a true coronal suture.

Whereas the pineal foramen and to a lesser extent the cartilage epiphyseal bar are potentially useful landmarks for inferring the relationship of the neural crest-mesoderm boundary to the coronal suture, lineage tracing in several model organisms provides more direct evidence (Figure 3). In mouse, use of a *Wnt1-Cre* allele to indelibly mark neural crest derivatives (Jiang et al., 2002), and a *Mesp1-Cre* allele to mark mesodermal derivatives (Yoshida et al., 2008), shows that the coronal suture forms at a clear boundary between the neural crest-derived frontal and mesoderm-derived parietal bones; the fibrous tissue of the suture itself is largely mesoderm-derived (Merrill et al., 2006). The situation in avians appears to be different (Figure 3). Quail-chick chimera experiments by Le Lièvre in 1978 (Le Lièvre, 1978) and Noden in 1982 (Noden, 1982) suggested only partial neural crest contribution to the frontal bone, although Couly et al. in 1993 (Couly et al., 1993) provided evidence that the frontal bone is entirely of neural crest origin. In 2006, Evans and Noden performed more precise labeling of both neural crest and mesoderm cells with retroviral injections and concluded that the frontal bone is of dual origin (Evans and Noden, 2006), with the ‘coronal suture’ at the border of the mesoderm portion of the frontal and the mesoderm-derived parietal bone. Similar to chick, lineage tracing of neural crest derivatives in zebrafish with a *Sox10*-Cre allele revealed a dual origin of the ‘frontal’ bone (the homolog of the mammalian parietal based on paleontological evidence), with the ‘coronal suture’ sitting at a mesoderm-mesoderm boundary (Kague et al., 2012; Mongera et al., 2013) (Figure 3). Lissamphibians show particularly wide variation of ‘coronal suture’ position relative to germ layer origins (Figure 3). Grafting of GFP-labeled neural crest or mesoderm in the Mexican axolotl (*Ambystoma mexicanum*) revealed the ‘coronal suture’ to be at a neural crest-mesoderm boundary, as in mice (Maddin et al., 2016), yet in the African clawed frog (*Xenopus laevis*) neural crest grafts suggest that the ‘coronal suture’ lies at a neural crest-neural crest boundary (Piekarski et al., 2014). This wide variation could, however, be related to metamorphosis as other skeletal traits are also highly divergent in modern lissamphibians . This variation in germ layer origins relative to the coronal suture, even within a clade of tetrapods such as that of lissamphibians, indicates that caution should be exercised in extrapolating germ layer boundaries in organisms for which experimental studies have not been conducted. For example, while it is a reasonable and parsimonious assumption that the human coronal suture sits at a neural crest-mesoderm boundary as in mouse, this needs to be formally tested.

The diverse positioning relative to germ layer origins is a complication when assigning homology to what is called the coronal suture across bony vertebrates (Schultze, 2008b). One solution is to define the coronal suture based on embryology in mouse, i.e. occurring at the anterior-posterior boundary of major neural crest-derived bones (the frontals) and major mesoderm-derived bones (the parietals). In this definition, the axolotl would have a true coronal suture. In chickens and zebrafish, the coronal suture would not be homologous to mouse, as it sits at a mesoderm-mesoderm boundary instead of a neural crest-mesoderm one. Hanken and colleagues proposed that the chick frontal bone (and by inference the zebrafish frontal bone) is equivalent to a fusion of the homologs of the murine neural crest-derived frontal and the anterior portion of the mesoderm-derived parietal bone, and hence should be termed ‘frontoparietal’. In such a scheme, the chick and zebrafish ‘parietal’ bones would represent the posterior portion of the murine parietal bone, or alternatively the equivalent of the mammalian postparietal or interparietal (Maddin et al., 2016). However, Fabbri et al. argued that the mesoderm-derived portion of the chicken frontal bone may correspond to remnants of a mesoderm-derived postorbital bone that fuses with the frontal early in development, with the postparietal being lost in modern avians (Smith-Paredes et al., 2018). If so, this would suggest a correspondence of the chicken and murine parietal bones, with fusion of the mesoderm-derived postorbital onto the posterior portion of the frontal creating a mesoderm-mesoderm interface at the coronal suture. In such a way, the chicken coronal suture could be considered a modified form of the mammalian coronal suture (Figure 3A). In contrast, using the pineal gland as a reference, the zebrafish coronal suture appears posteriorly shifted relative to both the mouse and chick coronal suture (Blümel et al., 2019), suggesting that the zebrafish ‘coronal’ is the homolog of the mammalian lambdoid suture as discussed earlier (Figure 3A). From an embryological perspective, the zebrafish ‘frontal’ bone may be better described as a ‘frontal-parietal’ fusion (reflecting neural crest and mesoderm contributions) and the ‘parietal’ as a ‘postparietal’ bone (Hirasawa and Kuratani, 2015).

Over the course of evolution, repositioning of the coronal suture would be expected to have major functional effects on head morphology, given that sutures serve as flexible joints that allow for transmission of biomechanical stress and as sites of directional bone growth (Esteve-Altava and Rasskin-Gutman, 2015). This re-positioning of sutures, due to fusions, divisions, and interpolations of bones, is a common theme across evolution. The adult human skull is composed of 22 bones, versus the 74 bones of the adult zebrafish skull, suggesting wide divergence of both species from their common ancestor circa 420 million years ago (Cubbage and Mabee, 1996; Hanken and Hall, 1993). Several examples of gain and loss of sutures and ossification centers have been recorded for extant vertebrates and in the fossil record (De Beer, 1937). For example, the mammalian interparietals, located posterior to the parietal bone, show extreme diversification in pattern. Lineage tracing in mouse shows that the interparietals are seamless fusions of medial neural crest-derived postparietal (Jiang et al., 2002) and lateral mesoderm-derived tabular bones (Yoshida et al., 2008), in much the same way that the chicken frontal and mammalian stapes are seamless fusions of neural crest- and mesoderm-derived bone (Thompson et al., 2012). From observing fetal or perinatal stages of over three hundred species, the interparietal bone was observed to be singular, as in the Japanese macaque; paired, as in the Amur hedgehog; tripartite, as in the bottle-nosed dolphin; or quadripartite, as in the cow. This varying number of interparietal bones can even be detected within a single species, as in the tree and rock hyraxes (Koyabu et al., 2012). These results suggest that interparietal bones are formed from the variable fusion of two neural crest-derived medial and two mesoderm-derived lateral ossification centers. The diversity of interparietal patterns indicates that sutures can be quite readily gained or lost in this region.

An important question then is whether there remains an embryonic remnant of two separate ossification centers in species where the ‘frontal bone’ is a composite of neural crest- and mesoderm-derived tissue. As discussed earlier, bird embryos transiently display separate ossification centers corresponding to frontals and postorbitals that later fuse into a single frontal bone. In their dinosaurian ancestors, however, the frontals and postorbitals remain separate through adulthood (Smith-Paredes et al., 2018). It was further hypothesized that fusion of the mesoderm-derived postorbital onto the neural crest-derived frontal, which created a new mesoderm-mesoderm coronal suture boundary, may have facilitated forebrain expansion along the dinosaur-avian lineage. In contrast, imaging of *RUNX2*:GFP+ pre-osteoblasts in zebrafish revealed only a single ossification center for the frontal bone, indicating that neural crest- and mesoderm-derived tissues intermix before the start of ossification (Kague et al., 2016). This may be analogous to the mammalian stapes, where only a single cartilage condensation is recorded despite its mixed neural crest and mesoderm origin (Thompson et al., 2012). These findings suggest different ontogenies for the mesoderm-mesoderm boundary of the ‘coronal suture’ in chicken versus zebrafish, reinforcing the idea that the chicken and zebrafish coronal sutures are not homologous despite their similar mesoderm-mesoderm interfaces.

In summary, lineage-tracing experiments demonstrate that the mouse and zebrafish coronal sutures are positioned at dissimilar tissue-origin boundaries, in agreement with analyses of comparative morphology and the fossil record. While it may be tempting to initially categorize ‘coronal sutures’ at similar tissue boundaries (e.g. mesoderm-mesoderm in chick and zebrafish) to be homologous, knowledge of the evolutionary and developmental formation of these structures pushes us to more accurately hypothesize historical connections and thus homology. Notably, we propose that the chick coronal suture may be a modified homolog of the mouse coronal suture, whereas the zebrafish coronal suture is homologous to the mouse lambdoid suture. It also becomes apparent that skull bones appear (e.g. tetrapodomorph frontal, coelacanth posterior parietal), disappear (e.g. chicken postparietal), and fuse (e.g. chicken frontal and postorbital) frequently during evolution, complicating the assignment of suture homology across vertebrates.

## New insights into brain-skull signaling in positioning homologous sutures

Whereas assignment of homology based on paleontological criteria relies on evolutionary continuity, homology based on embryological criteria could be based on germ layer origins and/or the source of inductive signaling (Hirasawa and Kuratani, 2015). For example, it has been proposed that limb buds represent deep, serial homologs of the pharyngeal arches (but see Rui and Molnar, 2014). This is based on topology and similarities of the signaling molecules produced by the epithelium, despite the skeletogenic mesenchyme of the limbs being mesoderm-derived and the arches being neural crest-derived (Gillis and Hall, 2016; Shubin et al., 2009). This idea of using molecular signals to deduce deep homology has also been proposed in drawing parallels between tetrapod and arthropod limbs (Panganiban et al., 1997), as well as cephalopod limbs (Tarazona et al., 2019), structures normally thought of as analogous but not homologous. In the skull, neural crest- and mesoderm-derived mesenchyme can seamlessly integrate into an ossification center, and the anterior-posterior limit of these germ layers may vary across vertebrate evolution. It may therefore be more informative to define suture homology based on the sources of inductive signals in the embryonic skull, rather than the germ layer origins of the connective tissues. In theory, there could be a limited number of signaling centers in the embryonic head capable of inducing sutures, with the germ layer origins of the mesenchyme being independent from the inductive signals. In such a scheme, sutures would be considered homologous across species if they were induced by equivalent signaling centers.

**Figure 4.**
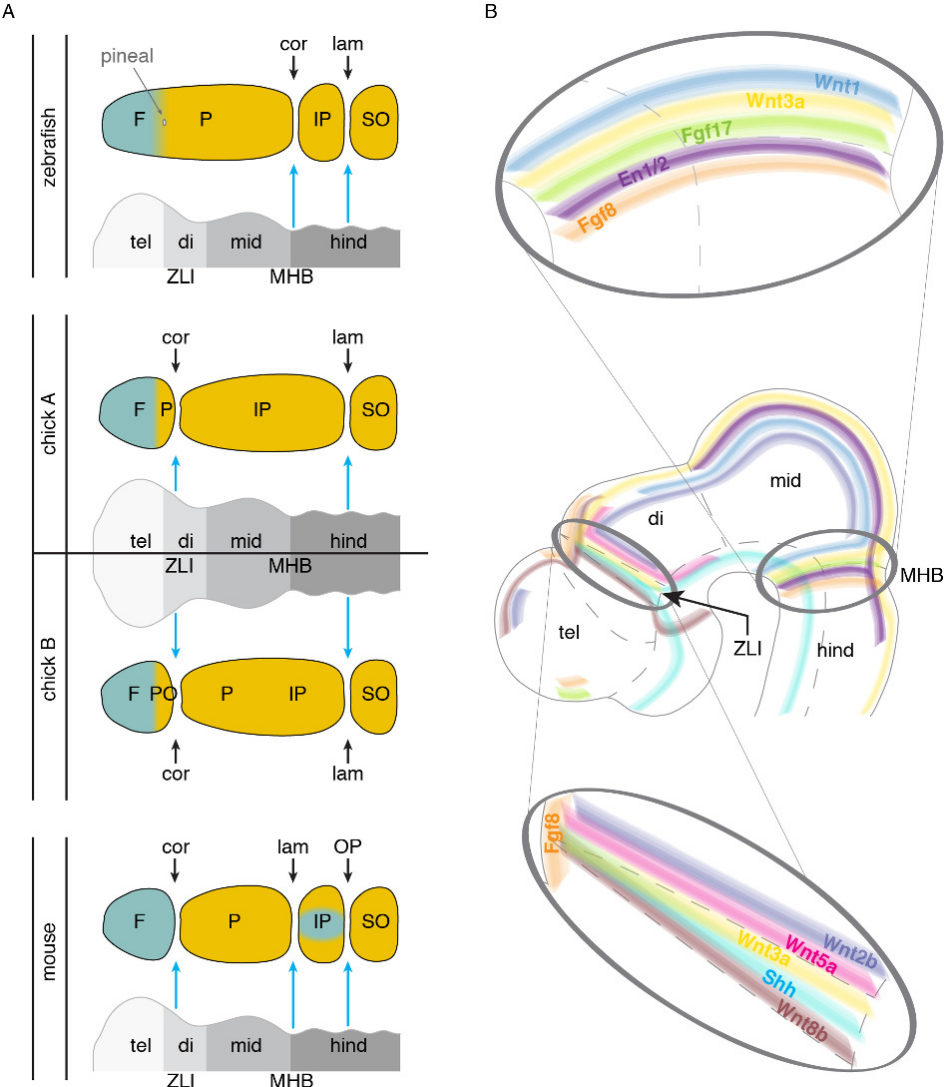
Models of brain to skull interactions across vertebrates. (**A**) Cranial bones are depicted relative to embryonic brain, with crest-derived bone in teal and mesoderm-derived bone in yellow. Comparison of zebrafish, chick, and mouse proposes the zona limitans intrathalamica (ZLI) and midbrain-hindbrain boundary (MHB, or isthmic organizer) as major signaling centers for suture development. For chick, two interpretations are shown: ‘chick A’ in which the mesoderm-derived portion of the ‘frontal’ bone is homologous to the anterior part of the mammalian parietal, and ‘chick B’ in which the mesoderm-derived postorbital is fused to the frontal. (**B**) Signaling molecules enriched at the ZLI and MHB include Wnts, Fgfs, En1/2, and Shh, all of which have known roles in proper skull and suture formation. F, frontal; P, parietal; IP, interparietal; SO, supraoccipital; PO, postorbital; tel, telencephalon; di, diencephalon; mid, midbrain; hind, hindbrain; ZLI, zona limitans intrathalamica; MHB, midbrain-hindbrain boundary; cor, coronal suture; lam, lambdoid suture; OP, occipitointerparietal suture.

The embryonic brain is an attractive candidate for providing such suture-inducing signals, as it is in intimate association with the overlying mesenchyme that will form the bony calvarium that protects it. In addition, the embryonic brain is subdivided by signaling centers, such as at the telencephalon-diencephalon, forebrain-midbrain, and midbrain-hindbrain boundaries, and these are rich in signaling molecules known to be important in controlling osteogenesis. In the late 1960s, Schowing examined the role of the chicken brain in skull bone development, finding, for example, that excision of the midbrain and hindbrain resulted in loss of the parietal bone (Schowing, 1968). More recently, Fabbri et al. evaluated skull morphology across numerous reptiles and birds and noted that the frontal bone is invariably found above the forebrain, and the parietal bone above the midbrain (Fabbri et al., 2017). In a variety of mammals, the coronal suture aligns with the middle of the forebrain (Blümel et al., 2019), roughly where the telencephalon-diencephalon boundary and pineal gland develop (Sood, 1980), and molecular analysis in mouse supports alignment of the coronal suture with the telencephalon-diencephalon boundary (Deckelbaum et al., 2012). The lambdoid suture, which separates the parietal and interparietal bones of mouse, is located just anterior to the cerebellum, a structure derived from the midbrain-hindbrain isthmus (Papp et al., 2015). We suggest a model in which signals from the telencephalon-diencephalon boundary (also known as the zona limitans intrathalamica, or ZLI) specify the mammalian, and possibly the avian, coronal suture in part by keeping the frontal and parietal bone anlagen separate (Figure 4A). More posteriorly, the mid-hindbrain boundary (MHB) would perform an analogous role in specifying the murine lambdoid suture, and by implication the zebrafish coronal suture if we consider it homologous to the murine lambdoid suture (Figure 4A).

It is important to note that the relationships between the developing brain and sutures have not been experimentally addressed in sufficient detail in any species (though see Schowing, 1968). These relationships may also change dramatically during development, such as in humans where the forebrain undergoes massive expansion, thus making it difficult to pinpoint embryonic stages where correlations between brain boundary and suture positions are informative. Further, the embryonic source of suture mesenchyme remains controversial. In mouse, lineage tracing with an *En1*-CreER allele at E10.5 (Deckelbaum et al., 2012), as well as DiI labeling (Ting et al., 2009), suggests active migration of mesenchyme from the supraorbital ridge to the future suture. Alternatively, the same progenitors that mediate the growth of the calvarial bones from their leading edges may contribute to the sutural stem cells that feed continued calvarial expansion. If the developing brain does play a role, it could act transiently and locally to block osteogenic differentiation, thus ensuring that separate ossification centers form. Or it could function more continuously to prevent osteogenic differentiation either in progenitors at the bone fronts and/or in future suture mesenchyme cells. For the coronal suture, any inductive role from the brain would likely be transient and operate at initial stages of frontal and parietal bone specification. Consistent with this, conditional deletion of one copy of *Twist1* in the mesoderm or neural crest of mouse can shift the location of the coronal suture due to tissue-autonomous acceleration of parietal or frontal bone growth, respectively (Teng et al., 2018). As presumably the brain itself would not be affected in conditional mutants (in particular for mesodermal *Twist1* deletion), this would argue against a continuous role for brain signaling in maintaining the coronal suture throughout development.

At a molecular level, brain boundaries are signaling centers that express several types of factors implicated in bone and suture development (Figure 4B). Fgf8 and Fgf17 are present at or near the ZLI and MHB (Martinez-Ferre and Martinez, 2012; Xu et al., 1999), and Fgf8 is required for development of the forebrain-midbrain (Scholpp et al., 2003) and midbrain-hindbrain (Lee et al., 1997) boundaries. Given that osteogenic mesenchyme sits above the brain during these early stages, it is conceivable that Fgfs could signal to the mesenchyme to control osteogenic differentiation and/or pre-specify sutural cells. Loss of the coronal suture is seen in several human syndromes caused by mutations in Fgf receptors: Pfeiffer (*FGFR1*), Apert (*FGFR2*), Beare-Stevenson (*FGFR2*), Crouzon (*FGFR2*), and Muenke (*FGFR3*) (Twigg and Wilkie, 2015). In further support of a dual role for Fgf signaling in brain and suture specification, expression of *Engrailed1* and *2* (*En1/2*) become progressively restricted to the midbrain-hindbrain boundary in chick, frog, and mouse (Lee et al., 1997; Davis et al., 1991; Gardner and Barald, 1991; Marin and Puelles, 1994), and Fgf8 has been shown to induce *En2* expression (Lee et al., 1997). At similar stages, *En1* is expressed in supraorbital ridge mesenchyme near the ZLI prior to calvarial bone development (Figure 4B), and lineage tracing of these *En1^+^* cells revealed contribution to the coronal suture mesenchyme (Deckelbaum et al., 2012).

Wnt signaling has also been implicated in suture development, with *Wnt2b*, *Wnt5a*, and *Wnt8b* expressed at or bordering the ZLI (Martinez-Ferre and Martinez, 2012), *Wnt1* at the MHB, and *Wnt3a* at both the ZLI and MHB (Dorsky et al., 1998; Quinlan et al., 2009) (Figure 4B). The negative Wnt regulator *Axin2* is also prominently expressed at the coronal suture of mice (Yu et al., 2005). Mutations in mouse *Axin2* (Behr et al., 2013) and human *AXIN2* (Yilmaz et al., 2018) have been reported to cause craniosynostosis, although the metopic and sagittal sutures and not the coronal are most commonly affected by mutations in these orthologous genes.

It is possible that distinct brain organizer centers produce similar suites of signaling factors (e.g. Fgfs and Wnts) to promote suture formation, with the ability of the mesenchyme to respond to a particular brain organizer varying between species. For example, the ZLI could induce a coronal suture in mouse and chick but not zebrafish, and the MHB a lambdoid suture in mouse and coronal suture in zebrafish (Figure 4A). Alternatively, brain organizers may have distinct suture-promoting activities that could relate to distinct properties and genetic sensitivities of particular sutures. Of note, *Shh* is expressed at the ZLI but not the MHB (Jeong et al., 2011) (Figure 4B), suggesting that differential Hh activity might help explain sensitivities of the coronal versus lambdoid suture to genetic perturbations. Identifying whether specific brain regions induce the coronal and other sutures in diverse vertebrates, and the signals that they use, will be informative for assigning homology of sutures across species, as well as understanding how abnormal brain-skull signaling may affect development of particular sutures. Intriguingly, a role for brain signaling in patterning the skull may not be limited to the calvarium, as a recent report suggests that signals from the brain and olfactory epithelium pattern the nasal cartilage that in turn helps shape facial intramembranous bones (Kaucka et al., 2018).

## Role of cartilage in inhibiting bony sutures in development and disease

Why might some animals have a suture at a particular position in the skull, for example relative to a brain landmark, and others not? Absence of a suture could be due to lack of distinct ossification centers at initial stages (such as for the zebrafish coronal), to a failure to maintain distinct ossification centers (such as for the chick frontal-postorbital interface), or to later bone fusion (such as for many forms of human craniosynostosis). Possibly not by chance, the presence of cartilage is correlated with an absence of a suture in a number of contexts. In humans, the closure of sutures at puberty coincides with endochondral ossification, i.e. cartilage-templated bone formation, between the fusing bones (Pritchard et al., 1956). Similarly, normal closure of the mouse posterior frontal suture is accompanied by a cartilage intermediate (Sahar et al., 2005), and loss of the coronal suture in mice with gain-of-function Pdgfra signaling is accompanied by ectopic cartilage between the frontal and parietal bones (He and Soriano, 2017).

Cartilage is not only associated with closure of sutures during normal development and disease, but also with the absence of a suture during early embryological stages. As stated earlier, paleontological and embryological data argue that zebrafish lack a comparable suture at the position of the mammalian coronal suture, i.e. at the interface of neural crest and mesoderm germ layers and at the approximate position of the telencephalon-diencephalon boundary. Whereas zebrafish do not develop a suture at this location, there is a prominent cartilage rod, termed the epiphyseal bar, which runs across the entire roof of the skull at the boundary of neural crest- and mesoderm-derived mesenchyme (Kague et al., 2016). In contrast, at the neural crest-mesoderm boundary in mouse, only short cartilage bars, termed the tectum transversum, are seen at both the left and right supraorbital ridges; these do not connect across the skull roof (Percival and Richtsmeier, 2017). Further, the epiphyseal bar is maintained through adulthood in zebrafish, yet the tectum transversum is largely resorbed by adult stages in mouse. One possibility is that the full-length cartilage bar in zebrafish prevents the formation of a suture in the analogous position to where the mouse coronal suture forms. Perhaps, the telencephalon-diencephalon signaling center induces a prominent cartilage bar in zebrafish but not mouse, which then prevents a suture forming in zebrafish in the adjacent mesenchyme. As separate ossification centers are never seen in zebrafish, the epiphyseal bar cartilage would have to act before osteogenesis to prevent establishment of distinct bone centers and hence a suture. Such a model is supported by analysis of one of the earliest known teleost fishes, the Mesozoic *Leptolepis*, which similarly lacks a suture where the epiphyseal bar cartilage has left a groove in the skull (Patterson, 1975). Further investigation into the correlation between the presence of an epiphyseal cartilage bar and the lack of a coronal suture at the neural crest-mesoderm boundary in a variety of extant species will be needed, as well as functional experiments in model organisms testing the requirement for cartilage in preventing formation of a suture at that position.

## Commonalities between evolutionary changes in and pathological losses of sutures

What might the study of pathological loss of sutures in craniosynostosis tell us about potential developmental mechanisms by which sutures could change during evolution? Work in various craniosynostosis models reveals that suture loss can occur at multiple stages of development. In postnatal mice, ablation of the *Gli1*+ sutural stem cells that fuel continued bone growth results in a complete loss of all sutures (Zhao et al., 2015). In contrast, an earlier embryonic defect in frontal and parietal bone growth may be the cause of the later loss of the coronal suture in mice and zebrafish harboring mutations in homologs of *TWIST1* and *TCF12* (Teng et al., 2018). Finally, loss of the coronal suture in *Fuz* mutant mice is reflected by a failure to form separate frontal and parietal bone ossification centers (Tabler et al., 2016). Other studies have shown that breakdown of the boundary between the fibrous suture and the adjacent osteogenic mesenchyme is associated with suture loss (Merrill et al., 2006; Ting et al., 2009; Yen et al., 2010). For example, Eph-ephrin signaling has well known roles in establishing tissue boundaries in a number of organs (Cayuso et al., 2015), and mutations in *EFNB1* result in coronal synostosis in humans (Twigg et al., 2004). In mouse, the ligands ephrin-A2 and ephrin-A4 are expressed in cells overlying the coronal suture region and prospective frontal bone, in what is termed the ectocranial layer, and the receptor EphA4 is expressed in a complementary pattern within the frontal bone and coronal suture (Merrill et al., 2006). In *Epha4* mutant mice, inappropriate cell mixing was observed across the neural crest-mesoderm boundary where the coronal suture forms (Ting et al., 2009). These studies suggest that, in pathological situations, sutures can be lost by many different means.

A recurrent theme is the high degree of specificity in the types of sutures lost in different forms of craniosynostosis, particularly in syndromic forms. A case in point is Saethre-Chotzen syndrome, in which the coronal suture is preferentially affected (Twigg and Wilkie, 2015). Unexpectedly, mutations in homologs of the Saethre-Chotzen genes *TWIST1* and *TCF12* in mouse and zebrafish result in similarly specific loss of the coronal suture, despite the zebrafish coronal suture likely being homologous to the mouse lambdoid suture (Teng et al., 2018). The common phenotypes in mammals and zebrafish therefore suggest a similar developmental process at play, despite lack of homology of the suture affected. Formation of a suture requires early signaling to suppress osteogenesis and hence divide an early osteogenic field into separate bones. Perhaps, similar signals come from distinct sources along the anterior-posterior axis (e.g. the ZLI for the mouse coronal and the MHB for the zebrafish coronal). In contrast, the sagittal and metopic sutures arise from frontal and parietal bones on the right and left sides of the skull, with these growing large distances to meet along the dorsal midline at the roof of the skull; hence, the types of early bone-suppressing activities required to set up sutures at anterior-posterior junctions (i.e. coronal/lambdoid) would not be required for sutures such as the metopic/sagittal that form from widely separated bone anlagen. The requirement to more actively separate bone anlagen along the anterior-posterior axis might provide one explanation for why the non-homologous coronal sutures of mammals and zebrafish are similarly sensitive to mutations in genes such as *TWIST1* and *TCF12* that regulate bone progenitor biology. While the coronal but not lambdoid suture is affected in humans and mouse models of Saethre-Chotzen syndrome, in which *TWIST1* and *TCF12* mutations are heterozygous, only homozygous loss of both *twist1b* and *tcf12* disrupts the zebrafish ‘coronal’ (i.e. lambdoid homolog), potentially consistent with greater sensitivity of the coronal than lambdoid to *TWIST1/TCF12* disruption. In addition, whereas many markers of bone progenitors and suture stem cells appear to be common across sutures (Zhao et al., 2015; Maruyama et al., 2016), there is evidence for some molecular differences between sutures. For example, expression of *Shh* and *Ptc* has been reported to be high in the metopic and sagittal but low in the coronal suture (Kim et al., 1998), which could underlie differential sensitivities of the coronal suture to genetic changes. The distinct genetic sensitivities of sutures to pathological mutations might also suggest mechanisms whereby alterations of these same genes could drive changes in particular sutures during vertebrate skull evolution, and reciprocally why sutures that have shifted the most during evolution, such as the coronal, are the most sensitive to genetic perturbations in the human population. It will therefore be worthwhile to determine whether variation in the regulatory or coding regions of craniosynostosis genes are linked to the loss or gain of sutures across evolution.

## Future directions

We have learned much from human disease and mutant model organisms about how sutures can be lost, yet we still know little about how the individual bones of the skull, and hence the sutures that separate them, are positioned during embryogenesis (for a more general discussion of how development, evolution, and disease may be linked see Diogo et al., 2015 and Shubin, 2009). In contrast to the limbs, where the number and organization of endochondral bones is conserved across vertebrate evolution, there is much wider variation for the intramembranous bones of the skullcap. An important question for further investigation is the extent to which individualization of ossification centers is an autonomous property of the skeletogenic mesenchyme (such as for joint spacing in the zebrafish fin (Rolland-Lagan et al., 2012) and digits of tetrapods Hartmann and Tabin, 2001), and/or is controlled by non-autonomous signaling from the underlying brain or other embryonic structures. When distinct ossification centers arise, it is also not understood why some fuse early in development (e.g. the frontals and postorbitals of birds), at postnatal stages (e.g. the posterior frontals of mouse), or not at all (e.g. the skull bones of zebrafish). Is suture closure pre-programmed at an early stage in the cells that will contribute to the suture mesenchyme, or does it arise through later inductive signaling? In addition, why are some sutures selectively disrupted in genetic mutants? Answers to these questions will not only illuminate mechanisms of suture-specific craniosynostosis, and potential strategies to better treat particular syndromes, but may also give insights into how patterns of sutures can change over evolution as a fundamental aspect of the diversification of head morphology.

Advances in genetics and microscopy should aid in developing a better map of how skull bones are precisely positioned within the embryo. The recent development of optically controlled Cre systems should allow researchers to precisely label small groups of cells and follow their bone and suture fate long-term (Kawano et al., 2016; Sinha et al., 2010). The zebrafish system is particularly appealing for such experiments because of its optical clarity and external development, providing easy implementation of new imaging techniques. Combined with genetic experiments focused on manipulating potential signaling from the brain and other tissues, these studies should begin to unravel the logic by which individual osteogenic centers are specified and then kept separate over time. By more precisely defining the temporal and spatial nature of suture specification cues, we will be closer to understanding how changes in these cues underlie pathological and evolutionary changes in suture pattern. Along with technical advances in model organisms, a more comprehensive analysis of skull formation in diverse vertebrates would inform general principles by which sutures and/or ossification centers may be gained or lost. It is an exciting time for such ‘evo-devo’ research. For example, CRISPR/Cas9 technology is allowing researchers to assess gene function and create knock-in transgenic reporters for diverse vertebrate species not previously accessible for genetic engineering. By combining sophisticated embryological research in model organisms with comparative anatomy and targeted experimentation in non-model organisms, we will better understand the plasticity of neural crest and mesoderm contributions to the embryonic skull, and how conserved signaling centers specify sutures across diverse vertebrates.

Our analysis of skull bone evolution exemplifies the complexities in assigning homologies over distant evolutionary time. The skull in particular has greatly diversified in vertebrates, in part because changes in the jaws, brain, and sensory organs underlie key feeding and predation adaptations and constraints in evolution. Hence, one must be cautious in inferring homology based solely on simple markers based on the relative positions of the bones in the skull, as in the case of the mammalian ‘frontal’ and bony fish ‘parietal’ that are similarly located between the orbits. Careful consideration of independent landmarks, such as the pineal gland, across a broad series of transitional animals allows a better appreciation of evolutionary continuity, although even in this approach gaps in the fossil record and possible shifts in the landmarks themselves require further examination. Genetic evidence can also be confounding, as non-homologous structures can share biological processes – this is strikingly illustrated by the common loss of the mouse coronal and the zebrafish lambdoid-equivalent ‘coronal’ suture upon genetic perturbation of Twist1 and Tcf12. Resolving skull bone homology will require a better understanding of the shared developmental mechanisms driving their formation, as well as a more robust fossil record for all portions of the vertebrate tree of life. Principles learned from studying homology within the skull should also more broadly inform how patterning mechanisms and germ layer contributions can shift over deep evolutionary time to give rise to vertebrate novelties such as the limbs and jaws.
